# Inducing Apoptosis of Cancer Cells Using Sea Pen *Virgularia gustaviana *Extract Which is Comparable to Cembrane Diterpene Sarcophine

**Published:** 2018

**Authors:** Sharareh Sharifi, Pargol Ghavam Mostafavi, Ali Mashinchian Moradi, Mohammad Hadi Givianrad, Hassan Niknejad

**Affiliations:** a *Department of Marine Biology, Science and Research Branch, Islamic Azad University, Tehran, Iran.*; b *Department of Marine Chemistry, Science and Research Branch, Islamic Azad University, Tehran, Iran.*; c *Department of Pharmacology, School of Medicine, Shahid Beheshti University of Medical Sciences, Tehran, Iran. *; d *Medical Nanotechnology and Tissue Engineering Research Center, Shahid Beheshti University of Medical Sciences, Tehran, Iran.*

**Keywords:** Cembrane diterpene, Cancer cells, Apoptosis, Caspase-3, Caspase-8, Virgularia gustaviana

## Abstract

Marine Soft corals have frequently been studied in recent years because of their specific chemical compounds in tissue engineering and regenerative medicine. The aim of this study was to investigate anti-cancer property of extracted compound from *Virgularia gustaviana *and their effect on inducing of apoptosis. The extraction process was carried out with ethyl acetate for 5 days and the extract was separated by silica-gel column chromatography. The column was washed with n-hexane-ethyl acetate solvent at ratio of 10:0 to 0:10. Thin layer chromatography (TLC), High performance thin layer chromatography (HPTLC), high performance liquid chromatography (HPLC), and 13C NMR spectroscopy were used for qualitative identification of compounds. The viability of HeLa and MDA-MB-231 cancer cells was investigated using MTT assay at the concentrations of 25, 50, and 100 µL/mL of extracted compounds. Immunocytochemistry and Western Blot analyses were used to evaluate expression of apoptotic markers caspase-3 and caspase-8 in cancer cells after treating with effective fractions (based on viability of cancer cells) and the results were compared with Sarcophine. From ten isolated fractions (A-J), Retention time and Retention Factors (Rf) of fractions G, I, and J were the same as Sarcophine. Fraction G, I, and J dose-dependently decreased cancer cell viability compared to control group and Sarcophine. Treatment of cancer cells with the latest fraction increased expression of caspase-3 and caspase-8 demonstrating induction of apoptosis as possible mechanism of action. According to the results, the compounds extracted from *Virgularia gustaviana *inhibit the growth of cancer cells by inducing of apoptosis pathway; an effect which needs to be further investigated in the future studies.

## Introduction

In spite of increasing advances in prevention and treatment, cancer is one of the major causes of death in the world (1). Clinical treatment of cancer is currently relied mostly on surgery, chemotherapy, and radiotherapy. As these methods are not effective in all cases, novel therapeutic approaches have been developed in recent years including immune therapies, efficient drug delivery systems, and applying natural bio-molecules (2, 3).

Several studies have reported the extraction of natural bio-molecules from marine sources with the capability of preventing tumour growth (4). Interestingly, some of these marine bio-molecules have been approved for anti-cancer purposes in clinic (5). 

Among marine organisms, marine sessile invertebrate are a rich sources for obtaining bioactive natural molecules (6). Owing to lack of physical defensive mechanisms, sessile invertebrates have developed chemical defence system including secretion of unique secondary metabolites into their surrounding environment with protective roles and also helpful for competing behaviours with other species (7, 8). A majority of the last decade studies about sessile invertebrate *Pennatulacea *(as an order of class *Anthozoa*) focused on the potential of *Pennatulacea* derived bioactive molecules in treating neoplasm (7). The order of *Pennatulacea *contains sea pens, which are colonial marine invertebrates including 300 species. The sea pens have feather-like appearance available at intertidal zones (15 meter) up to 600 meters depths (9). Marine *Anthozoa *are attracted great attentions in light of their structural diversity and wide range of their biologically active metabolites (10). 

Terpenoids are organic compounds extracted from *Octocoralina, *as a subclass of* Anthozoa*, which demonstrate anti-cancer effects (7). Terpenoids are classified to Hemiterpenes, Monoterpenes, Sesquiterpenoids, Diterpenoids, Sesterterpenes, Triterpenoids, Tetraterpenoids, and Polyterpenoid (11). Sarcophine (C_20_H_28_O_3_), as a Diterpene with high anti-tumour activity (9), has the capability to induce apoptosis in cancer cells both *in-vitro* and *in-vivo*, as well as showing anti-inflammatory effects (12).

The aim of this study was to extract, characterize, and evaluate anti-cancer effects of *Virgularia gustaviana* (a genus in *Octocorallina* subclass) extract and compare result with that of Sarcophine. Therefore, we first obtained the ethyl-acetate extract of *Virgularia gustaviana *and then characterized the separated fractions using chromatography and compared the data with Sarcophine characterization results. To evaluate anti-cancer effects of extracted chemical fractions and Sarcophine, their cytotoxic effects on HeLa and MDA-MB-231cancer cell lines were measured. Moreover, the expression of caspase-3 and caspase-8 (as apoptosis markers) was assessed following treatment of cancer cells with extracted chemical fractions and Sarcophine. 

## Experimental

*Animal material*

Marine sea pen *Virgularia gustaviana *was collected by patrolling on the intertidal zone of the estuary Sura in Bandar Abbas city, south of Iran (Persian Gulf). The collected animals were immediately frozen, transferred to the lab and kept in -20 °C.

*Obtaining ethyl acetate extract and separation of chemical fractions*

In order to obtain organic extract, the frozen sea pen (1 kg, wet weight) was chopped into small pieces and freeze-dried (13). Then, ethyl acetate (EtOAc) solution (Merck, Germany) was added to the powdered samples and the resultant mixture was shaken for three days in room temperature (three separate amount of ethyl acetate were added up to totally 2 litters). After that, the resultant ethyl acetate extract was filtered using Whatman filter paper (125 mm), and the organic phase of filtered solution was extracted using distilled water (14). Finally, the solvent was removed by evaporation using rotary evaporator. In the next step, the crud extract of sea pen was subjected to column chromatography on silica gel column (6 × 120 cm, 230–400 mesh; Merck, Germany) and eluted with *n*-hexane-EtOAc (gradient separation 10:0 to 0:10) (15). The result of column chromatography was 10 different fractions (labelled from A to J), primarily characterized by thin layer chromatography (TLC) method using pre-coated Kieselgel 60 F254 (0.25 mm, Merck, Germany) (12). Fraction G (2.5 g) was subjected to silica gel flash column chromatography using Hexane-Dichloromethane-Methanol (1:1:0.5), as a mobile phase. Sub fraction of G6 was collected and re-crystallized.

Sub fraction G6: white powder; mp 62.9 °C; 1H NMR (CDCl3, 400 MHz) δ: 2.34 (2H, t, J = 8.0 Hz, CH2), 1.63 (2H, t, J = 8.0 Hz, CH2), 1.26 (s, -(CH2)n-), 0.87 (3H, t, J = 8.0 Hz, CH3). DEPT-Q (CDCl3, 100 MHz) δ: 14.11 (CH3), 22.69 (CH2), 24.70 (CH2), 29.07 (CH2), 29.24 (CH2), 29.36 (CH2), 29.44 (CH2), 29.59 (CH2), 29.65 (CH2), 29.67 (CH2), 29.68 (CH2), 31.92 (CH2), 34.05 (CH2), 179.60 (q).

The other fractions and sub fractions need purification, so biological test such as MTT assay and immunocytochemistry and also chemical evaluations of fractions G, I, and J was assessed in this study. 

*High performance thin layer chromatography (HPTLC)*

HPTLC is a method, which is usually employed to obtain a chemical fingerprint of a natural compound. The samples were applied on HPTLC plate (Silica gel 60 F 254 glass 20 × 10 cm, Merck, Germany), and the EtOAc-n-hexane (1:10) was used as mobile phase. Fifty µL of each fraction was loaded in plates. Commercially available Sarcophine (Abcam, UK) was used as positive control (1 µL sample). The resultant bands were detected by vanillin immersion and sulphuric acid spraying followed by heating to 120 °C for 5 min. 

*High performance liquid chromatography (HPLC)*

The retention time of each fraction and commercial Sarcophine was measured using HPLC (Cecile, UK) and normal phase column (Hibar 250 × 10 mm, silica gel 60, 5 μm, Merck, Germany). The best results were obtained by utilizing the mobile phase consisting Methanol-Deionized water (pH 3.5 adjusted with phosphoric acid) with 60:40 v/v ratio, and flow rate of 1.5 mL/min. The highest absorbance was in 220 nm (16).

*Cell culture and Cytotoxicity assay*

The cytotoxic effect of each fraction was evaluated using HeLa and MDA-MB-231 cancer cell lines. The cells were cultured in RPMI 1640 (Sigma, USA) supplemented with 10% fetal bovine serum (Sigma, USA) and 1% penicillin/streptomycin (Sigma, USA), and incubated in humidified air containing 5% CO_2 _at 37 °C (17). After reaching 75% confluence, the cells were detached using Trypsin/EDTA and sub-cultured in 24-well plates (5 × 10^4 ^cell/well) for 24 h in 5% CO_2 _at 37 °C overnight. To evaluate the cytotoxic effect of each sample, 1 mg of each fraction wasdissolved in 1 mL dimethyl sulfoxide (DMSO, Merck, Germany), and diluted with RPMI (1:9 v/v). Then, the prepared solution from each fraction was added in concentration of 25, 50, and 100 µL for each well. The cells cultured without treatment and the cells treated with Sarcophine were considered as negative and positive controls, respectively. The plates were incubated in 5% CO_2 _at 37 °C for 24 h until cytotoxicity assay was performed (18).

The viability of cancer cells was investigated using 3-(4,5-dimethylthiazol-2-yl)-2,5-diphenyl tetrazolium bromide (MTT) assay. MTT solution (5 mg of MTT/mL of distilled water) was filtered and added to each well (40 µL/well). Then, the plates were incubated for 4 h in 5% CO_2 _at 37 °C. After that, the formosan crystals were dissolved in 900 µL DMSO per each well. Finally, the optical absorbance was measured at 570 nm using spectrophotometer (CE7500; Cecil, Cambridge, UK) (17). 

*Immunocytochemistry*

The expressions of caspase-3 and caspase-8 (apoptosis markers) in cancer cells treated with 100 µL of fractions G, I, and J were evaluated using immunocytochemistry. We have chosen the cells treated with these three fractions due to their highest cytotoxicity among obtained fractions. To perform immunocytochemistry, the cells were fixed with 2.5% paraformaldehyde for 30 min at room temperature followed by several washing with PBS. Then the samples were incubated with 10% bovine serum albumin (Sigma-Aldrich) for 30 min. Next, permeabilization of the fixed cells was achieved by using 0.2% Triton X-100 (Sigma-Aldrich) for 30 min. After that, the cells were incubated with primary monoclonal antibodies including anti-caspase 3 antibody (1:300, Cell Signalling, USA) and anti-caspase 8 antibody (1:100, Cell Signalling, USA) in humidified environment at 4 °C for an overnight. After washing with cold PBS, the secondary goat anti-rabbit FITC antibody (1:500, Abcam) was applied for 2 h at room temperature (18, 19). Isotype-matched rabbit IgG antibody was applied instead of primary antibodies as negative controls. The nuclei of cells were also stained by 4,6- diamino-2-phenylindole (Sigma-Aldrich) (18). 

For quantitative analysis of expressing caspase-3 and caspase-8 in treated cells, the number of positive cells in each sample was counted using computerized software (ImageJ, National Institutes of Health, Bethesda, Maryland).

*Western blot analysis*

HeLa and MDA-MB-231 cells were lysed in lysis buffer containing 125 mM Tris-HCL (pH 6.8), 10% v/v β-Mercaptoethanol, 10% glycerol, 4% SDS, and trace amount of bromophenol blue. 

Depending on protein concentration (measured based on Bradford assay), the cell lysates were diluted in RIPA buffer to the gel-loading concentration of proteins (2.5 μg/μL), mixed with equal volumes of sample buffer. Protein samples were separated using a protein electrophoresis. The proteins separated by SDS-PAGE were blotted onto nitrocellulose membrane for 3 h using a blotting apparatus (Bio-Rad). The membrane was blocked with 5% non-fat dry milk or 5% BSA in TBS for 20 min and incubated with the primary antibodies anti-caspase 8 antibody (1:100, Cell Signaling, USA) and anti-caspase 3 antibody (1:300, Cell Signaling, USA) at 4 °C overnight. After the incubation, the membrane was washed three times (5 min each time) with TBS containing 0.1% Tween-20. Then, it was incubated for 2 h with the corresponding horseradish peroxidase-conjugated secondary antibody (Santa Cruz Biotechnology, Santa Cruz, CA, USA). The membrane was finally washed and the band signals of the interesting proteins were normalized by Beta actin (ab8227 Abcam) and Chemiluminescence signal was detected. The relative band intensities of blots were measured using the ImageJ software. 

*Statistical analysis*

All data were expressed as mean ± standard error of mean. The statistical analysis was performed using one-way analysis of variance (one-way ANOVA) with Tukey post-test. A *P*-value less than 0.05 was considered statistically significant. 

## Results

**Figure 1:**
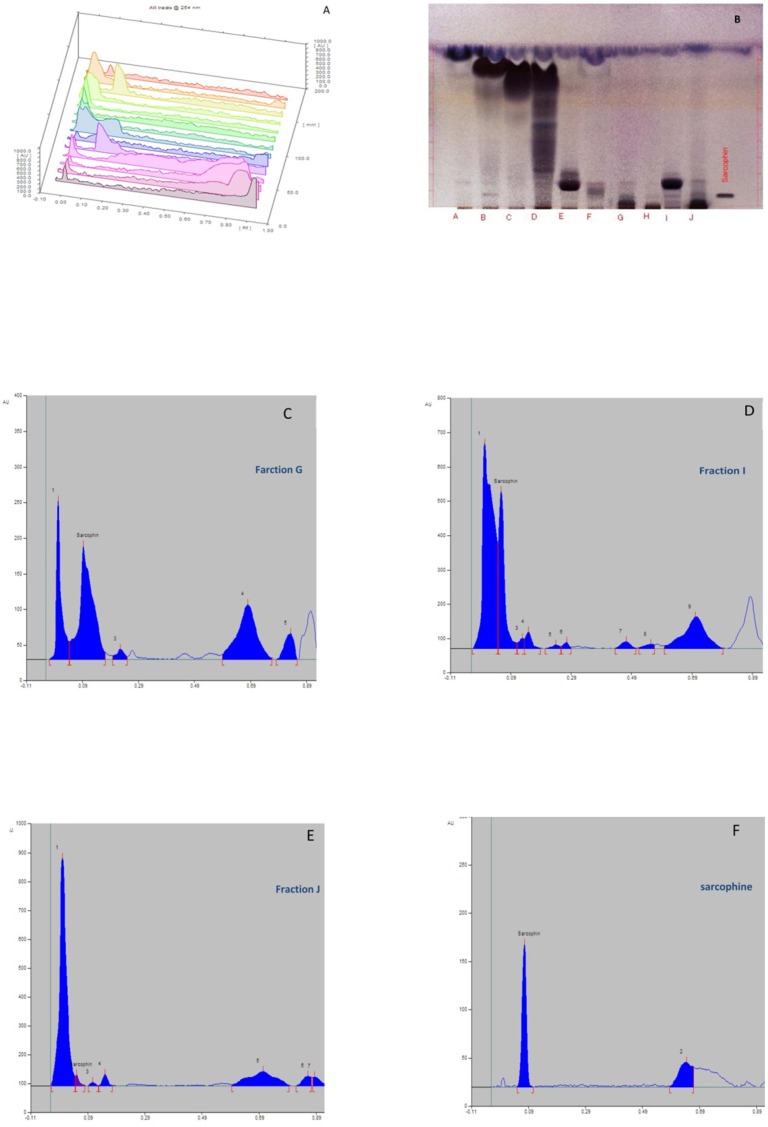
A) Feather-like appearance of Sea pen* Virgularia gustaviana.* (B-G) Representative images from HPTLC results. (B) three–dimensional chromatogram of extracted fractions in a wavelength of 254 nm; (C) HPTLC plate in which showed band of Sarcophine and fractions A-J; (D) Chromatogram belong to three-dimensional image of fraction G; (E) Chromatogram belong to three-dimensional image of fraction I; (F) Chromatogram belong to three-dimensional image of fraction J; (G) Chromatogram belong to three-dimensional image of Sarcophine

**Figure 2:**
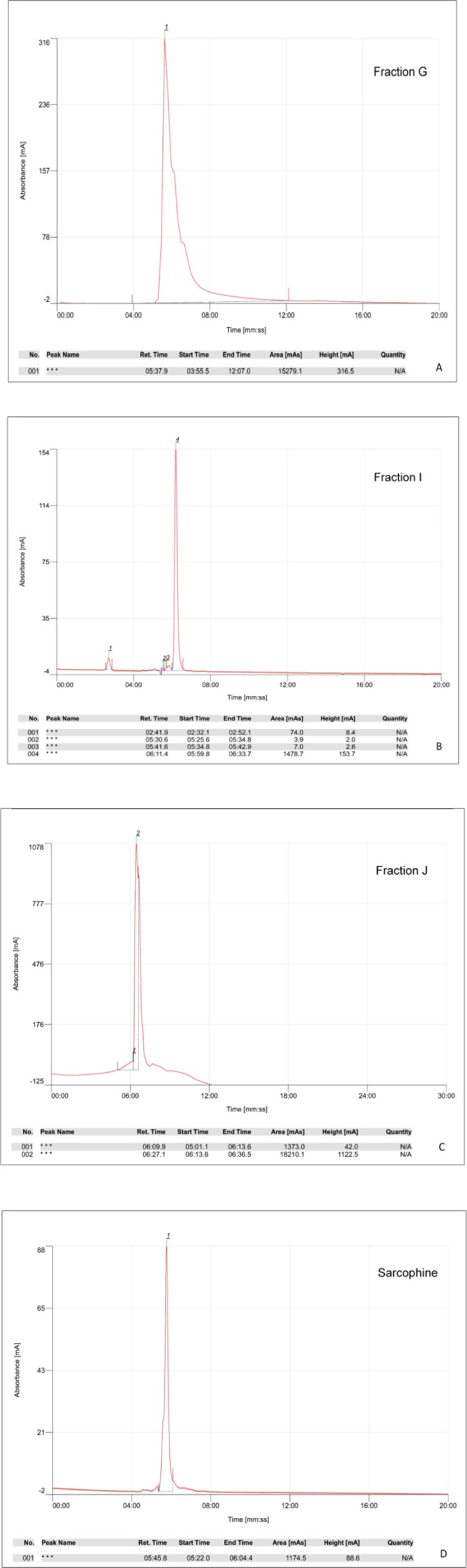
HPLC chromatograms of 20 µL injection of fraction G, I, J and Sarcophine which showed in panels A, B, C and D, respectively

The ethyl acetate extract of Sea pens *Virgularia gustaviana *was isolated using silica gel column chromatography. According to the results of thin layer chromatography, totally 10 chemical fractions were obtained. HPTLC method was performed to achieve a chemical fingerprint of each fraction compared with Sarcophine. The results of HPTLC showed that obtained fractions G, I, and J have similar characteristics with Sarcophine (Figures 1A-F). These fractions and Sarcophine were also characterized using high-pressure liquid chromatography (HPLC). Analysis of the fractions G, I, and J using HPLC showed that there are some compounds in each of these fractions that have near retention times with Sarcophine. The characterization results revealed that fractions G, I, and J may contain some compounds with a close relation to Sarcophine (Figure 2).

The structures of the purified fraction G was determined on the basis of 1D and 2D NMR spectra and by comparison of their spectral data with those of the literature (20, 21). The isolated compound from fraction G was identified as compound Hexadecanoic acid. Structural elucidation of the isolated compound was based on 1H NMR, 13C NMR spectroscopic data.

**Figure 3:**
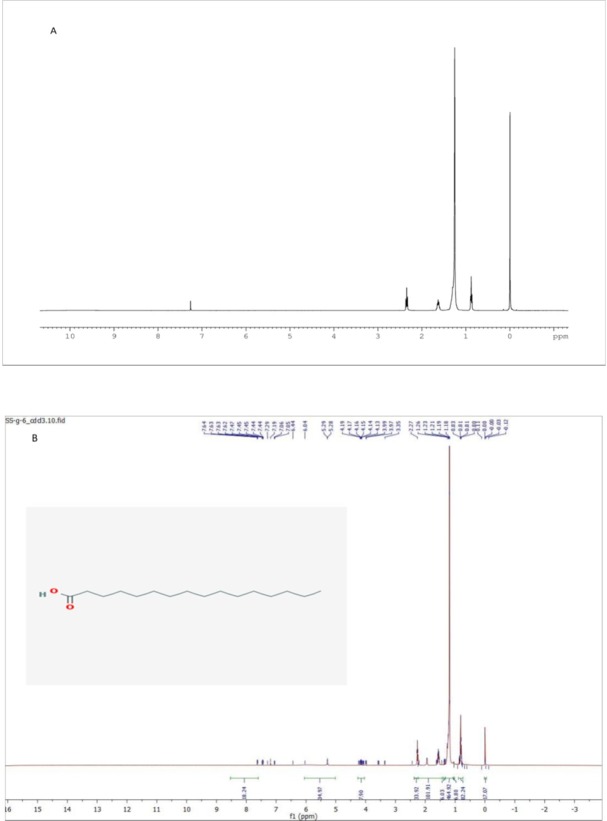
*A)* *1D* *HNMR of Hexadecanoic (B) 1D HNMR and Structure of Hexadecanoic acid from sea pen *Virgularia gustaviana

Sub fraction G6: molecular formula (C_16_H_32_O_2) _was obtained as white powder with melting point 62.9 °C. According to the 1 H, 13C, 2D NMR (H,H-COSY, HMQC, HMBC) experiments and also MS spectra and by comparing these spectroscopic data with those reported in the literatures, this compound was assigned to be Hexadecanoic acid (Figure 3).

**Figure 4.**
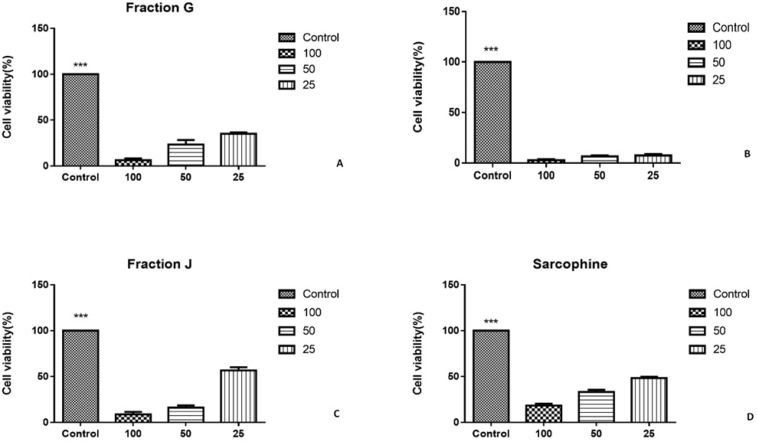
A) Viability of HeLa cancer cells evaluated by MTT assay after 24-h incubation with fractions obtained from sea pen species *Virgularia gustaviana* and commercial Sarcophine. HeLa cancer cells treated by fraction G. (B) fraction I was applied on HeLa cancer cells. (C) treated cell by fraction J. (D) cell viability of HeLa cancer cell after treatment with Sarcophine

**Figure 5:**
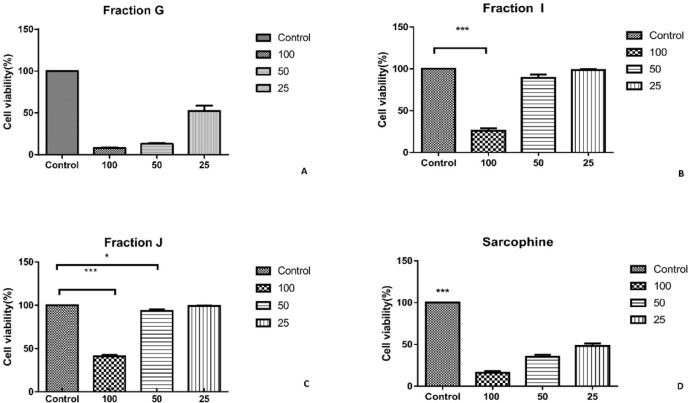
A) Viability of MDA-Mb-231cancer cells evaluated by MTT assay after 24 h incubation with fractions obtained from sea pen species *Virgularia gustaviana* and commercial Sarcophine. MDA-Mb-231cancer Cells treated by fraction G. (B) Fraction I was applied on MDA-Mb-231 cancer cells. (C) Treated cancer cell by fraction J and (D) cell viability of MDA-Mb-231 cancer cell after treatment with Sarcophine

As the characterization results showed that the fractions G, I, and J obtained from ethyl acetate extract of sea pen *Virgularia gustaviana *may have similar components to Sarcophine, the cytotoxic effects of these three fractions and Sarcophine were evaluated on HeLa and MDA-MB-231 cancer cell lines using MTT assay (12). Each prepared fraction was added in cell culture media in concentrations of 25, 50, and 100 µL. The MTT assay results after 24 h revealed that 100 µL of fractions G, I, J, and Sarcophine reduced the viability of HeLa cells to 6.33 ± 2.2%, 8.96 ± 2.53%, 2.80 ± 0.87%, and 29.00 ± 3.6%, respectively. Fraction G and I dose-dependently reduced cell viability of HeLa cells (Figure 4). Moreover, the viability of MDA-MB-231 cancer cells was reduced to 8.00 ± 0.3% after 24-h incubation with 100 µL of fraction G. The viability of later cells also decreased to 41 ± 1.52% and 26.00 ± 3.05 following treatment of cells with 100 µL of fractions J and I, respectively. Sarcophine (100 µL) displayed cytotoxic effect on the MDA-MB-231 which reduced viability to 16.00 ± 1.15% (Figure 5).

**Figure 6:**
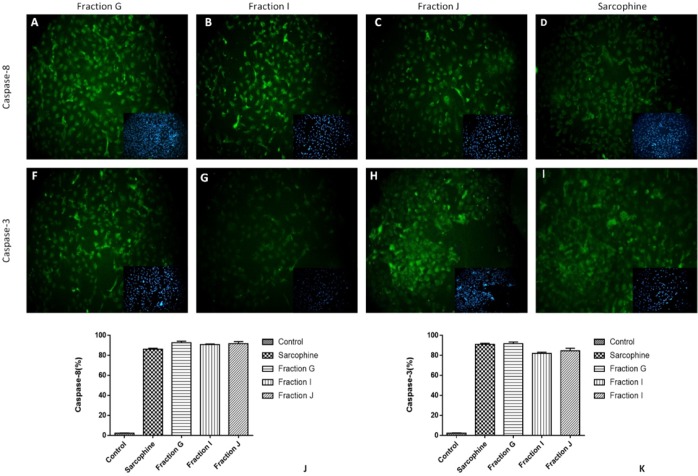
Representative images of immunocytochemistry against Caspase-8 and Caspase-3 in HeLa cancer cell after treatment by 100 µL of fractions G, J, I and Sarcophine. The inset in each figure shows nuclei staining by DAPI. Quantitative analyses of immunocytochemistry images demonstrated that the effect of fractions G, I, and J in inducing apoptosis and expression of caspase-8 (J) and caspase-3 (K) was comparable with Sarcophine

To evaluate the underlying mechanism of inducing cancer cell death via obtained fractions and Sarcophine, the expression of caspase-3 and caspase-8 was measured using immunocytochemistry. After treatment of both HeLa and MDA-MB-231 cells with 100 µL samples of fractions G, I, J, and Sarcophine, the immunocytochemistry results demonstrated that the expressions of both caspase-3 and caspase-8 increased, leading to apoptosis of cells. After treatments with fraction G, J, I, and Sarcophine, approximately 90%, 87%, 90%, and 90% of HeLa cells expressed caspase-3, respectively. Moreover, the expression of caspase-8 was induced in approximately 95%, 95%, 88%, and 58% of HeLa cells treated with fraction G, I, J, and Sarcophine (Figure 6).

**Figure 7:**
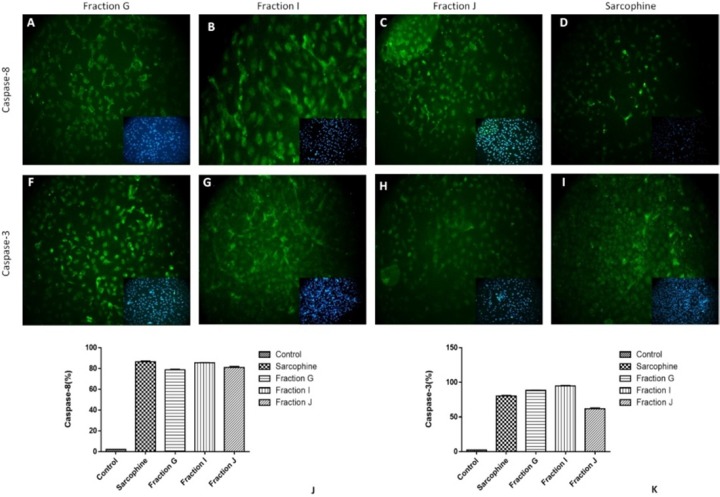
Representative images of immunocytochemistry against of caspase-8 and Caspase-3 in MDA-Mb-231 cancer cell after treatment with 100 µL of fractions G, J, I and Sarcophine. The inset in each figure shows nuclei staining by DAPI. (J and K) Quantitative analyses of immunocytochemistry images demonstrated that the effect of fractions G, I, and J in inducing apoptosis and expression of caspase-8 and caspase-3 was comparable with Sarcophine

We also evaluate the effect of the obtained fractions and Sarcophine on expression of caspase-3 and caspase-8 in MDA-MB-231 breast cancer cells. The results were similar to HeLa cells. Seventy-eight percent of MDA-MB-231 cells expressed caspase-8 after 24-h incubation with faction G. The percentage of MDA-MB-231 cells which expressed caspase-8 was 80% and 90% following treatment with fractions J and I, respectively. Sarcophine induced the expression of caspase-8 in 86% of MDA-MB-231 cells. In addition, caspase-3 was expressed in 88%, 96%, 60%, and 80% of MDA-MB-231 cancer cells treated with fractions G, I, J, and Sarcophine, respectively (Figure 7). These results demonstrate that the fractions obtained from ethyl acetate extract of sea pen *Virgularia gustaviana *induce apoptosis in cancer cells.

**Figure 8:**
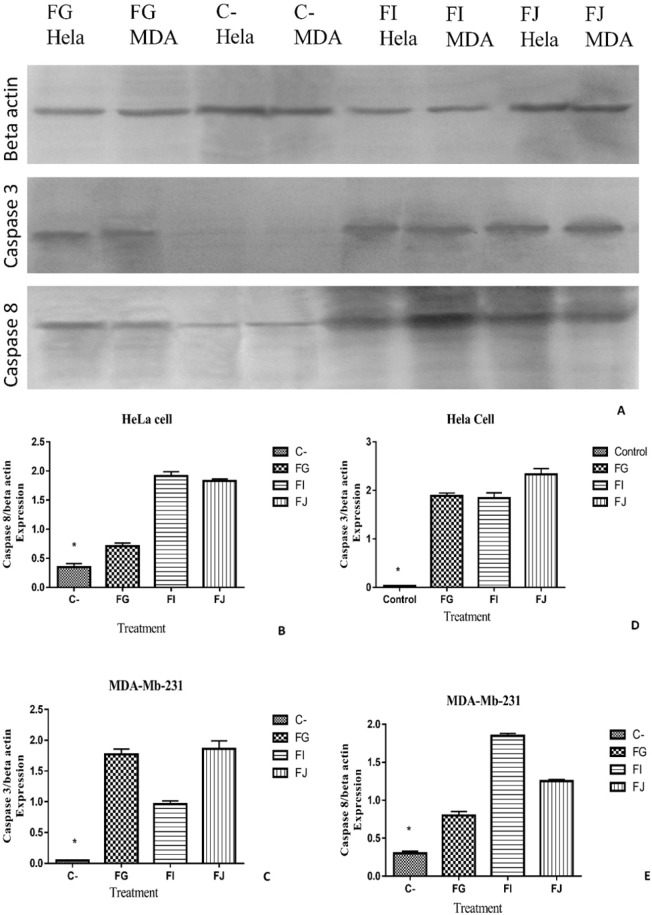
Representative image of Western blot analysis in which showed that expression of Caspase-8 and Caspase-3 was increased in HeLa and MDA-Mb-231 cancer cell lines after treatment with fractions G, I and J. (C-): Control group, (*) : *P*-value less than 0.05

To further explore the induction of apoptosis as mechanism of action of sea pen (*Virgularia* *gustavina*) fractions on HeLa and MDA-Mb-231 cancer cells, the expressions of apoptotic proteins were evaluated by western blot analysis. As shown in Figure 8, expression of caspase-3 and caspase-8 were elevated after treatment by fractions G, I, and J in both cancer cell lines HeLa and MDA-MB-231.

## Discussion

Marine biotechnology is a new emerging field for approaching to new therapeutic strategies (22). Active biomolecules extracted from marines are employed as new drugs for treatment of various diseases *e.g.* cancers, infectious diseases, inflammatory and neurological disorders, and treatment of ulcers. Sea pens are rich sources of active bio-molecules such as Terpenoids, Steroids, and Alkenes (23). It has been reported that 61% of studies about biomolecules of sea pen focused on Terpenoids (7). 

In this study, chemistry and biology of the bioactive metabolites of marine sea pen *Virgularia gustaviana* was assessed by separation and isolation techniques. Further biological, toxicological, and immunocytochemistry techniques were examined.

In this research, 10 chemical fractions were isolated from ethyl acetate extracts of sea pen* Virgularia gustaviana* and the result of HPLC and HPTLC analyses of obtain fractions showed that fractions G, I, and J probably belong to Terpenoids family. Consistent with this study, it has been shown that a great deal of compounds extracted from ethyl acetate procedure is Diterpene. Junceol A, a new Sesquiterpene and two known Diterpenoids, Sclerophytin A, and Cladiellisin were extracted from *Virgularia juncea,* by ethyl acetate extraction (24)*. *Briarenolides F and G as a new briarane Diterpene from *Briarum Sp. *were also obtained from ethyl acetate separation (25). The other polar solvents were also employed to isolate Diterpenes from sea pen. A cembrane diterpene from sea pen *Gyrophyllum sibogae *and Braiaran Diterpene Anthoptilides A-E from sea pen *Anthoptilum *cf. *kukenthali* were extracted by 2-propanol and dichloromethane, respectively (26, 27)*. *

From HPLC and HPTLC, it seems that fraction G, I, and J contain Sarcophine or the other derivatives of Sarcophine family. Sarcophine as a Cembrane Diterpene isolated from *Sarcophyton* glaucum is one of the essential chemical bioactive compound. The results of the qualitative identification of compounds by comparing them shows that Sarcophine or other derivatives of this category probably exist in fractions G, I, and J obtained from sea pen *Virgularia gustaviana. *However, more studies are needed to accurately identify compounds responsible for anti-cancer property of sea pen extracted compound.

In this study, the isolated fractions and also Sarcophine were applied to cancer cells MDA-MB-231 and HeLa with concentrations of 25, 50, and 100 µL. From 10 isolated fractions only 3 fractions G, I, and J demonstrated anti-proliferative activity against cancer cells. Similar to Sarcophine, fraction G, I, and J concentration-dependently decreased the viability of both cancer cell lines. The amount of effects in reduction of cell viability by fractions G, I, and J was comparable with Sarcophine. 

One of the most important advantages of Sarcophine is induction of apoptosis as anti-proliferative mechanism of action. From anti-cancer view, apoptosis is superior to necrosis since it does not induce inflammation which might damage to healthy tissues and organs. Apoptosis as a programmed cell death is divided into two pathways including the extrinsic and intrinsic pathways. The extrinsic pathway relies on the receptor and initiates apoptosis by activating caspase-8, while the intrinsic pathway occurs through the release of cytochrome c from mitochondria and commences the apoptosis by activating caspase-3 (17). Immunocytochemistry method was used to investigate apoptosis as possible mechanism for inducing cancer cells viability reduction by fractions G, I, and J. The results showed that fraction G, I, J, and Sarcophine induced apoptosis in cancer cells by expression of caspoase-3 and caspase-8. Increase of caspase-8 and caspase-3 expressions in HeLa cells by fraction G, I, and J were similar to Sarcophine. The same results achieved in MDA-Mb-231 cancer cell. Expression of caspase-8 and caspase-3 in MDA-MB-231 cancer cells treated by fractions G, I, and J increased which the results were comparable with Sarcophine. These results were consistent with previous studies in which Sarcophine was employed as cytotoxic agents against cancer cells. It has been shown that Sarcophine has potent cytotoxic effect on HL-60 and A431 human cancer cell lines through induction of apoptosis and DNA fragmentation (28). In the other study in which the effect of Sarcophine was compared with that of semisynthetic derivative of Sarcophine, the viability of MCF-7 and MDA-MB-231 were decreased by Sarcophine at low concentration, while semisynthetic agent has no effect on viability and apoptosis after 72 h on both cell lines (29).

The results of western blot analysis confirmed immunocytochemistry data and showed that the expression of caspase-3 and 8 were increased after treatment of HeLa and MDA-Mb-231 cells by fractions G, I, and J. The similar study was done on 13-acetoxysarcocrassolide extracted from Sarcophyton crassocaule which show that this compound reduced viability of bladder female transitional cancer (BFTC) cells by induction of apoptosis (30). The other compound Sinulariolide is a Cembrane Diterpene which induces apoptosis through the mitochondrial-related apoptosis pathway. Western blotting result similarly showed that the treatment of cancer cells by Sinulariolide increased expression of cleaved caspase-3 and cleaved caspase-9 (31). 

In this study, sub-fraction G-6 from fraction G was purified and compound Hexadecanoic acid was identified. Cytotoxic effect of fatty acid and sterol has been reported earlier for *Octocorallia* extracts. Cytotoxic polyhydroxysterol, 23, 24, -dimethylcholest-16(17)-E-en-3β, 5α, 6β, 20(S)-tetraol isolated from soft coral *Sarcophyton trocheliophorum* showed potent growth inhibitory activity against human HL60 leukemia, M14 skin melanoma, and MCF7 breast carcinoma cells with EC_50_ values of 2.8, 4.3, and 4.9 μg/mL, respectively (32). The fatty acids such as 14-methylpentadecenoic acid, hexadecenoic acid, 12-methyltetradecanoic acid and 14-methylhexadecanoic acid isolated from the *Streptomyces sp. *showed also significant inhibition against topoisomerase I activity (33). In addition, Lobophytosterol is a sterol from soft coral *Lobophytum laevigatum *which showed cytotoxic activity against HCT-116 , A549 and HL-60 cells with IC_50_ values of 3.2, 4.5, and 5.6 μM, respectively (34). 

From these promising results, three fractions extracted from sea pen demonstrate strong cytotoxic effects on human cervical cancer cell line HeLa and Breast cancer cell line MDA-Mb-231 in which the result were comparable with Cembrane Diterpene Sarcophine.

## Conclusion

In conclusion, characterization and biological experiments represented that there are similarities between Sarcophine and three fractions extracted from sea pen *Virgularia gustaviana*. In the future studies more extensive researches will be required to identify precisely compounds responsible for anti-cancer effect and to carry out in-vivo studies of extracted compounds.
